# Assessing risk factors associated with breakthrough early post-traumatic seizures in patients receiving phenytoin prophylaxis

**DOI:** 10.3389/fneur.2023.1329042

**Published:** 2024-01-04

**Authors:** Eugene Generoso, Calvin Diep, Carolyn Hua, Elizabeth Rader, Ran Ran, Nathanael J. Lee, Lucia Rivera-Lara

**Affiliations:** ^1^Department of Pharmacy, Stanford Health Care, Palo Alto, CA, United States; ^2^Department of Emergency and Critical Care Medicine, Oregon Health & Science University, Portland, OR, United States; ^3^Department of Neurology, Stanford Health Care, Palo Alto, CA, United States

**Keywords:** traumatic brain injury, post-traumatic epilepsy, phenytoin, risk factors, seizure prophylaxis

## Abstract

**Objective:**

Post-traumatic seizure (PTS) is a well-known complication of traumatic brain injury (TBI). The objective of this study was to identify risk factors associated with breakthrough early PTS in TBI patients receiving phenytoin prophylaxis.

**Methods:**

This was a single-centered retrospective study including adult patients admitted to the intensive care unit (ICU), had a TBI, and started on phenytoin for seizure prophylaxis within 24 h of admission. The primary outcome was the incidence and factors associated with early PTS, defined as a confirmed seizure on a continuous electroencephalogram within 7 days of TBI. Secondary outcomes included the association between early post-traumatic seizures and ICU length of stay, hospital length of stay, and in-hospital mortality.

**Results:**

A total of 105 patients were included in the final analysis. Patients with early PTS were older (65 vs. 48 years old, *p* = 0.01), had a higher Marshall score (5 vs. 2, *p* = 0.01), were more likely to have a Marshall score > 2 (73 vs. 37%, *p* = 0.01), and had more neurosurgeries for hematoma evacuation (57 vs. 19%, *p* = 0.01). In patients with early PTS, 57% had a level at the time of seizure, and of those, 87.5% had a therapeutic level (>10 mcg/mL). Patients with early PTS had a longer ICU length of stay (14.7 vs. 5.9 days, *p* = 0.04) and a greater proportion of hospital mortality (21 vs. 2%, *p* = 0.02).

**Conclusion:**

Patients with higher age, Marshall score, and neurosurgical procedures for hematoma evacuation had higher incidences of breakthrough early PTS despite the use of phenytoin prophylaxis. The majority of patients with early PTS had therapeutic phenytoin levels at the time of seizure when a level was available; however, approximately half (43%) did not have a level.

## 1 Introduction

Post-traumatic seizure (PTS) is a well-known complication of traumatic brain injury (TBI) resulting in long-term impairment, disability, and reduced quality of life (1, 2). Currently, there is mixed evidence and consensus regarding the safety and efficacy of phenytoin for PTS prophylaxis (3–6). The Brain Trauma Foundation guidelines recommend phenytoin for 7 days following severe TBI to prevent early seizures (within 7 days of injury; Level IIA recommendation) (3). Some trials have also shown no benefit and potential harm including longer hospital stays and worse functional outcomes at discharge with the use of antiepileptic drugs (AEDs) for early PTS prophylaxis, though many are limited by retrospective designs (7–11). Risk factors for early PTS include Glasgow Coma Scale (GCS) score of <10; immediate seizures; PTS amnesia lasting >30 min; linear or depressed skull fracture; penetrating head injury; subdural, epidural, or intracerebral hematoma; cortical contusion; or chronic alcoholism; however, these are often identified in patients not receiving prophylactic AEDs (12, 13). Finally, greater than 60% of early PTS occur despite having therapeutic phenytoin levels (7, 8). The purpose of this study was to identify factors associated with breakthrough early PTS in TBI patients receiving phenytoin for seizure prophylaxis. This study also aimed to describe phenytoin monitoring practices for PTS prophylaxis given the limited guidance in the literature.

## 2 Methods

### 2.1 Study design and study population

This was a single-center, retrospective study conducted at Stanford Health Care (SHC), an academic level 1 trauma center, between January 1, 2010 and March1, 2023. Patients were included if they were 18 years or older, admitted to the intensive care unit (ICU), had an International Classification of Diseases (ICD) 9 or 10 code for TBI, and started on phenytoin for seizure prophylaxis within 24 h of admission for at least 6 days or until the first hospital seizure. Patients were excluded if they had a history of seizure, were on AEDs prior to admission, received concomitant AEDs while on phenytoin prophylaxis, had a reported seizure without confirmation on continuous electroencephalogram (cEEG), transitioned to comfort care within 7 days of admission, incarcerated, or pregnant.

### 2.2 Ethical approval

This study protocol was approved by the Stanford Institutional Review Board (IRB no. 66917, approved September 2, 2022). This study was conducted in accordance with the ethical standards of the Stanford IRB and with the Helsinki Declaration of 1975. For this study, formal consent was not required.

### 2.3 Study outcomes and definitions

The primary outcome was to examine the incidence and factors associated with early PTS, defined as a confirmed seizure on cEEG within 7 days of TBI documented by a neurologist in the electronic medical record (EMR). Data were extracted via chart review of the EMR. Admission computerized tomography (CT) imaging was reviewed by a neuroradiologist and a neurologist, blinded to outcomes, to calculate the Marshall score (definitions in Supplementary material) (14). Electrographic seizure was defined as epileptiform discharges averaging >2.5 Hz for ≥10 s (>25 discharges in 10 s), or any pattern with definite evolution and lasting ≥10 s (15). Electrographic status epilepticus was defined as electrographic generalized seizures for ≥5 continuous minutes, and focal status epilepticus was defined as electrographic focal seizures for ≥10 continuous minutes. The cEEG was interpreted by the on-called epileptologist and was categorized based on documentation in the neurology notes. Secondary outcomes included the association between early PTS and ICU length of stay, hospital length of stay, and in-hospital mortality.

Severe TBI patients are primarily managed by the trauma/surgical ICU team in consultation with the neurology team when the primary team deems it necessary. The use of cEEG is generally not a standard practice for all TBI patients at SHC. Continuous EEG monitoring is often initiated once a seizure is suspected or for unexplained encephalopathy and is at the discretion of the consulting neurocritical care and neurology team.

Initiation and dosing of phenytoin for early PTS prophylaxis was at the discretion of the treating team. Phenytoin monitoring and dose adjustments were also managed by the treating team. Phenytoin levels (mcg/mL) were corrected for hypoalbuminemia and renal dysfunction using the closest albumin and serum creatinine result preceding the phenytoin level (corrected phenytoin = measured phenytoin level/[(adjustment × albumin, g/dL) + 0.1], adjustment = 0.275; in patients with creatinine clearance <20 mL/min, adjustment = 0.2) (16). Appropriately drawn phenytoin maintenance level was defined as a level drawn 6–8 h following a phenytoin dose to reflect a trough level (17, 18).

### 2.4 Statistical analysis

IBM SPSS Statistics 22 (IBM Analytics, Armonk, NY, United States) was used to perform all statistical analyses with a predefined significance level of 0.05 by two-tailed asymptotic or exact tests. Parametric continuous variables were compared using ANOVA. Non-parametric continuous variables were analyzed using the Mann–Whitney U-test, and categorical variables were analyzed using Pearson’s chi-squared test or Fisher’s exact test. The relationship between seizures and age, Marshall score, number of neurosurgical procedures, and hematoma evacuation were further explored using univariate binary logistic regression. The low event rate for seizures precluded a single large multivariate binary logistic regression. Instead, bivariate binary logistic regression was performed on seizures vs. hematoma evacuation and age as well as seizures vs. Marshall score > 2 and age.

## 3 Results

**Figure 1:**
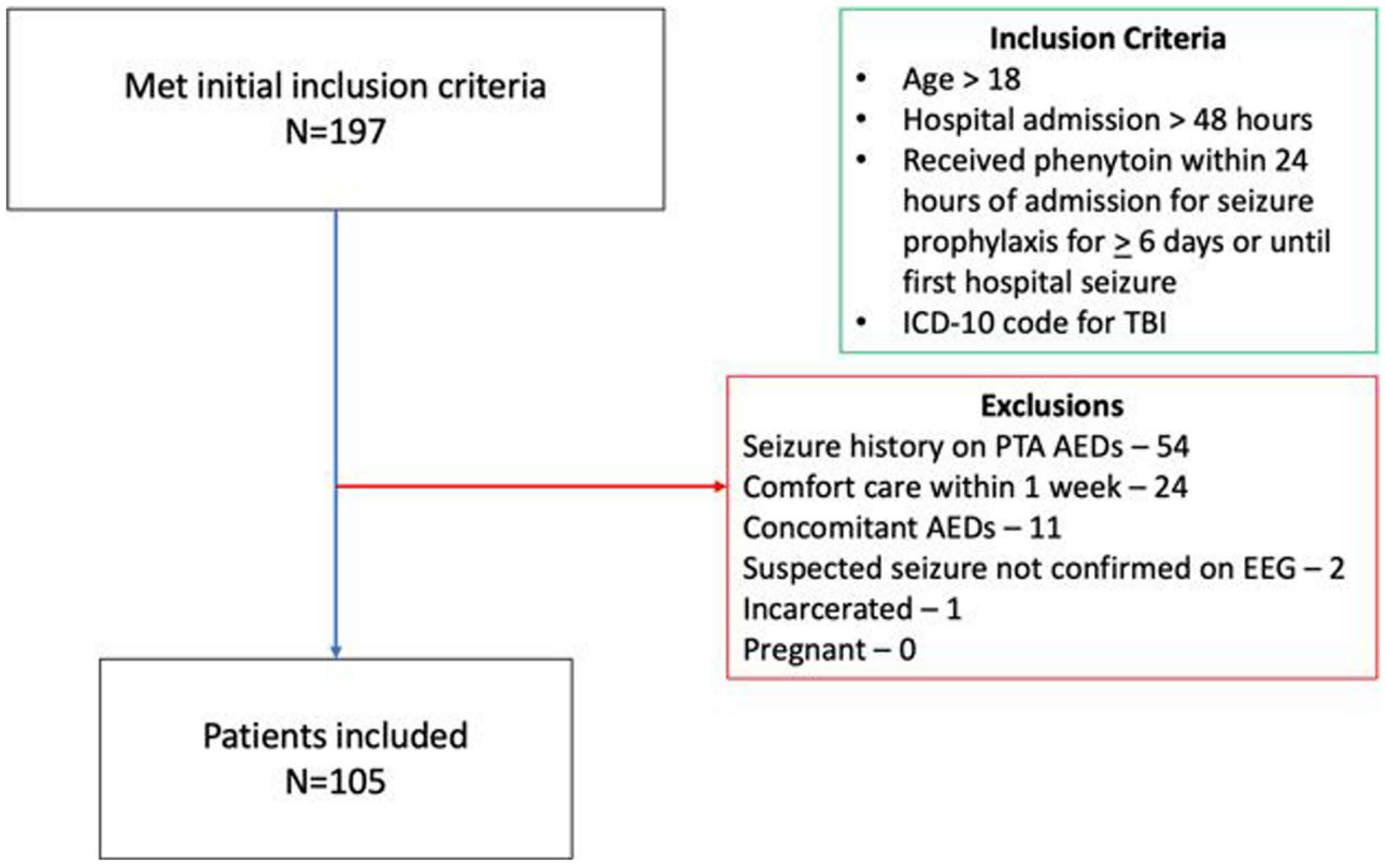
Patient inclusion/exclusion flow chart. ICD, International classification of diseases; TBI, Traumatic brain injury; PTA, Prior to admission; AED, Anti-epileptic drug; and EEG, Electroencephalogram.

A total of 197 patients were screened for inclusion. The most common reason for exclusion was seizure history on AEDs prior to admission (*n* = 54; see Figure 1). Of the remaining patients, 105 were included in the final analysis, 14 had early PTS, and 91 did not. Patients who had early PTS were older (65 vs. 48 years old, *p* = 0.01), had a higher Marshall score (5 vs. 2, *p* = 0.01), a greater proportion of patients with Marshall score > 2 (73 vs. 37%, *p* = 0.01), underwent more neurosurgeries for hematoma evacuation (57 vs. 19%, *p* = 0.01), and a higher number of neurosurgical procedures (1 vs. 0, *p* = 0.02). The average time from hospital admission to the first reported seizure on cEGG was 2.8 days in patients who had early PTS. Concomitant gabapentin use was more frequent in patients without early PTS (26 vs. 0%, *p* = 0.04). Patient characteristics are shown in Table 1.

**Table 1:** Patient characteristics for patients with and without early post-traumatic seizures.

	No seizure (*n* = 91)	Seizure (*n* = 14)	*p* value
Age (years), mean ± SD	48 ± 20	65 ± 21	**0.01**
Male, *n* (%)	68 (75%)	11 (79%)	1
BMI, mean ± SD	25 ± 7	24 ± 4	0.60
Admission GCS, median (IQR)	13 (7–14)	11.5 (8–14)	0.88
Marshall score, median (IQR)	2 (2–4)	5 (3–5)	**0.01**
Marshall score > 2, *n* (%)	34 (37)	11 (73)	**0.01**
ETOH ≥50 mg/dL, *n* (%)	17 (19%)	2 (14%)	1
Positive toxicology screen, *n* (%)	26 (29%)	4 (29%)	1
Amphetamine	6 (7%)	1 (7%)	1
Benzodiazepine	12 (13%)	1 (7%)	0.69
Cocaine	2 (2%)	1 (7%)	0.35
Opioids	8 (9%)	1 (7%)	1
THC	14 (15%)	1 (7%)	0.69
Mechanical ventilation, *n* (%)	59 (65%)	12 (80%)	0.22
Vasopressor use, *n* (%)	32 (35%)	8 (57%)	0.14
Hyperosmolar therapy, *n* (%)	52 (57%)	12 (86%)	0.07
Hypertonic saline	34 (37%)	9 (64%)	
Mannitol	2 (2%)	0 (0%)	
Both	16 (18%)	3 (21%)	
Concomitant injuries, *n* (%)	90 (99%)	14 (100%)	1
Subarachnoid hemorrhage	70 (77%)	9 (67%)	0.33
Subdural hematoma	54 (59%)	11 (79%)	0.24
Epidural hematoma	13 (14%)	1 (7%)	0.69
Intraventricular hemorrhage	14 (15%)	5 (36%)	0.13
Intraparenchymal hemorrhage	39 (43%)	7 (50%)	0.77
Depressed skull fracture	13 (14%)	1 (7%)	0.69
Cortical contusion	38 (42%)	6 (43%)	1
Diffuse axonal injury	10 (11%)	1 (7%)	1
Penetrating injury	1 (1%)	2 (14%)	0.05
Immediate seizure (within 24 h)	2 (2%)	1 (7%)	0.35
Number of concomitant injuries, median (IQR)	3 (2–4)	3 (3–4)	0.21
Number of neurosurgery procedures, median (IQR)	0 (0–1)	1 (1–2)	**0.02**
Neurosurgery procedure, *n* (%)	41 (45%)	11 (79%)	**0.02**
External ventricular drain	23 (25%)	4 (29%)	0.75
Bolt	4 (4%)	1 (7%)	0.52
Decompressive craniotomy	18 (20%)	5 (36%)	0.18
Hematoma evacuation	17 (19%)	8 (57%)	**0.01**
Prior to admission medications, *n* (%)			0.14
Antipsychotics	2 (2%)	1 (7%)	
Benzodiazepines	1 (1%)	0 (0%)	
Both	0 (0%)	1 (7%)	
Concomitant medications, *n* (%)			
Gabapentin	24 (26%)	0 (0%)	**0.04**
Scheduled benzodiazepine	8 (9%)	2 (14%)	0.62
Propofol infusion >48 h	17 (19%)	3 (21%)	0.73
Midazolam infusion >48 h	0 (0%)	1 (7%)	1
Time from admission to first seizure on cEEG (days), mean (SD)	NA	2.8 (1.6)	NA

BMI, Body mass index; ETOH, Ethanol; GCS, Glasgow Coma Scale; IQR, Interquartile range; SD, Standard deviation; THC, Tetrahydrocannabinol; cEEG, Continuous electroencephalogram; and NA, Not applicable. Bold values indicate statistically significant values *p* < 0.05.

Appropriately drawn phenytoin levels (level drawn 6–8 h after a phenytoin dose) were available in 55% of patients with no early PTS vs. 79% of patients with early PTS (*p* = 0.19). The duration of phenytoin prophylaxis was shorter in the early PTS group (2.5 vs. 7 days, *p* < 0.01). In patients with early PTS, 57% had a level at the time of seizure, and of those, 87.5% had a therapeutic level (>10 mcg/mL). Phenytoin level and dosing data are shown in Table 2.

**Table 2:** Phenytoin level and dosing data for patients with and without early posttraumatic seizures.

	No seizure (*n* = 91)	Seizure (*n* = 14)	*p* value
Phenytoin levels description, *n* (%)			0.19
*At least one appropriate* maintenance level drawn^a^	50 (55%)	11 (79%)	
Subtherapeutic	15 (16%)	3 (21%)	
Therapeutic/supratherapeutic^b^	35 (38%)	8 (57%)	
*N*o appropriate maintenance level drawn	21 (23%)	3 (21%)	
No maintenance level drawn	20 (22%)	0 (0%)	
Loading dose given, *n* (%)	67 (74%)	12 (86%)	0.51
Loading dose (mg/kg), mean ± SD	18.3 ± 5.3	17.2 ± 3.6	0.48
Initial total daily dose (mg), mean ± SD	300 ± 0	300 ± 0	1
Average total daily dose (mg), median (IQR)	300 (300–300)	300 (271–300)	0.59
Duration of phenytoin prophylaxis (days), median (IQR)	7 (6.55–7.85)	2.5 (1.6–4)	**<0.01**
Number of levels during first 7 days, median (IQR)			
Inappropriate levels	1 (0–4)	2 (1–3)	0.69
Appropriate levels	1 (0–2)	2 (1–3)	**0.04**
Dose titration, *n* (%)			0.27
None	71 (78%)	7 (50%)	
Increase	11 (12%)	4 (29%)	
Decrease	4 (4%)	3 (21%)	
Both increase/decrease	3 (3%)	0 (0%)	
Bolus	2 (2%)	0 (0%)	
Time to first therapeutic level (days), median (IQR)^c^	1.3 (0.7–2.7)	1.9 (1.4–4.3)	0.09
Average level during first 7 days (mcg/mL), mean ± SD	12.3 ± 5.6	13.2 ± 6.6	0.67
% time in therapeutic range, median (IQR)^d^	100 (0–100)	86 (33–100)	0.09
Phenytoin level at time of seizure^e^, *n* (%)			NA
No level	NA	6 (43%)	
Available level	NA	8 (57%)	
Subtherapeutic (< 10 mcg/mL)	NA	1 (12.5%)	
Therapeutic/supratherapeutic (≥ 10 mcg/mL)	NA	7 (87.5%)	

IQR, Interquartile range; SD, Standard deviation. ^a^Phenytoin levels were categorized as appropriately drawn if drawn 6–8 h after a dose. ^b^Patients were categorized as having therapeutic/supratherapeutic levels if greater than 50% of appropriately drawn levels within the first 7 days of injury were > 10 mcg/mL. ^c^Based on patients with > 1 therapeutic level: no seizure (*N* = 35); seizure (*N* = 8). ^d^Only for patients with > 1 appropriate maintenance level drawn; no seizure (*N* = 50), seizure (*N* = 14). ^e^Available level preceding seizure event. Bold values indicate statistically significant values *p* < 0.05.

Univariate regression analysis showed that the following were associated with early PTS: Age (odds ratio [OR] 1.04, 95% confidence interval [CI] 1.01–1.07, *p* = 0.01), Marshall score (OR 1.7, 95% CI 1.15–2.61, *p* = 0.02), Marshall score > 2 (OR 6.4, 95% CI 1.68–24.77, *p* = 0.01), and hematoma evacuation (OR 5.8, 95% CI 1.78–18.94, *p* = 0.02). Regression analyses assessing risk factors associated with early PTS are shown in Supplementary Tables 2–4.

The early PTS group had a longer ICU length of stay (14.7 vs. 5.9 days, *p* = 0.04) and a greater proportion of hospital mortality (21 vs. 2%, *p* = 0.02). Secondary outcomes are shown in Table 3. Of patients who experienced early PTS, 93% were focal and 7% were generalized seizures. Epileptiform activity on cEEG as noted by the neurology note was categorized as status epilepticus (36%), clinical (7%), or subclinical seizures (57%). Seizure data are shown in Supplementary Table 1.

**Table 3:** Secondary outcome results in patients with and without early post-traumatic seizures.

	No seizure (*n* = 91)	Seizure (*n* = 14)	*p* value
In hospital seizure after 7 days, *n* (%)	1 (1%)	0 (0%)	1
ICU length of stay (days), median (IQR)	5.9 (3.2–13.7)	14.7 (5–21.9)	0.04
Hospital length of stay (days), median (IQR)	11.9 (8.4–24)	20.6 (12.3–28.9)	0.09
Hospital mortality, *n* (%)	2 (2%)	3 (21%)	0.02

ICU, Intensive care unit; IQR, Interquartile range.

## 4 Discussion

In this study including critically ill patients with TBI receiving phenytoin for seizure prophylaxis, older age, higher Marshall score, Marshall score > 2, and neurosurgery for hematoma evacuation were associated with breakthrough early PTS. This study showed that patients with these characteristics seized despite receiving phenytoin prophylaxis and the majority having therapeutic phenytoin levels.

There are currently limited studies that investigate risk factors for breakthrough early PTS in patients receiving prophylactic AEDs. Majidi et al. (19) found that seizures occurred more frequently in patients with old age, African American ethnicity, moderate TBI, history of alcohol dependence, and subdural hematoma. However, the choice of AED used for PTS prophylaxis as well as the description of dosing and levels are limited. A *post-hoc* exploratory analysis found that hematoma evacuation and Marshall score > 2 were associated with early PTS occurrence after adjusting for age. Higher severity of injury (GCS < 10) and hematoma evacuation have also been found to be associated with early PTS in TBI patients not receiving pharmacologic seizure prophylaxis (12, 20, 21). Given the current literature and our findings, closer monitoring and aggressive AED dosing may be considered in patients with these characteristics.

Antiepileptic drug dosing and achieving effective target levels play a vital role in determining the efficacy of AEDs for preventing early PTS. There are limited recommendations regarding phenytoin monitoring as well as data on the relationship between therapeutic phenytoin levels and a rate of early PTS. A total serum phenytoin concentration of 10–20 mcg/mL is a widely accepted target range to prevent seizures, but this has not been confirmed (22). Retrospective and randomized studies in patients with severe TBI have reported that 60–100% of patients who experience early PTS have therapeutic phenytoin levels (7, 8). Similarly, 87.5% of patients in this study had a therapeutic phenytoin level when a level was available at the time of seizure occurrence. The high occurrence of early PTS despite having therapeutic phenytoin levels may indicate that the traditional target level of 10–20 mcg/mL is suboptimal and/or there is a subset of patients with a high epileptogenic focus that is not tempered by phenytoin alone. The average phenytoin level at the time of seizure was 17.1 mcg/mL. It is unclear whether alternative AEDs or multiple AEDs for these higher-risk patients would be effective. Based on the results of this study, higher vigilance in patients with these risk factors and possibly an alternative AED may be considered keeping in mind that efficacy is also unclear. Further studies are warranted to determine the optimal phenytoin target and AED for preventing early PTS as well as if there is a subgroup of severe TBI that will benefit more than others.

The incidence of early PTS after severe TBI while on prophylactic phenytoin ranges from 3 to 4% based on randomized and retrospective studies (5–7, 22–24). In this study, the incidence of early PTS was higher at 13%. These differences are likely due to the study population, severity of TBI, use of concomitant medications with anti-epileptic properties, phenytoin dosing, co-management of these patients with neurocritical care, and the use of a cEEG. This study only included patients admitted to the ICU with admissions >6 days; 99% had at least one type of brain injury on CT imaging, and 60% received hyperosmolar therapy. Based on the results of this study, patients with a Marshall score > 2, older age, and/or neurosurgical procedures for hematoma evacuation had higher incidences of breakthrough PTS; determining the optimal prophylactic AED regimen is key to reducing PTS incidences.

The other consideration is that AED prophylaxis could be ineffective for early PTS. Though guidelines recommend phenytoin for early seizure prophylaxis following severe TBI, there is currently mixed evidence regarding the efficacy of AEDs for preventing PTS. Temkin et al. found that phenytoin reduced the rate of early PTS compared to placebo (3.6 vs. 14.2%, *p* < 0.001) in a single-centered randomized trial in patients with severe TBI (5). Young et al. (7) found that phenytoin did not reduce the incidence of early PTS in patients with severe TBI with over 78% of patients with phenytoin levels >10 mcg/mL at days 1, 3, and 7 after injury. Several retrospective studies have also found no difference in the rate of early PTS in patients who received AED for prophylaxis including phenytoin, levetiracetam, or valproic acid compared to patients who received no AEDs (8–10). The mixed results could be attributed to the varying definitions of severe TBI across randomized trials as well as the varying severity of TBI included in retrospective studies. It is possible that prophylactic AED is ineffective as several studies suggest; however, there may be a subset of patients that may benefit that have yet to be identified.

There were several limitations to this study including its single-centered retrospective nature and the potential confounders inherent to such a design. Overall, 22% of patients did not have maintenance phenytoin levels drawn and 22% of patients had levels drawn inappropriately. In addition, in patients with early PTS, 43% did not have a level at the time of seizure and it is possible that these patients had subtherapeutic levels which may have led to seizure occurrence. However, this reflects a real-world practice as levels may not be drawn correctly or not drawn at all. Free phenytoin levels may be more accurate in the acute care setting. However, free phenytoin level is a send out lab with a 3-day turnaround time and is not commonly used for phenytoin monitoring at SHC. Instead, corrected phenytoin levels were used and reported, which is a common alternative when free phenytoin levels are not readily available. It is also difficult to pinpoint the relationship between seizure occurrence and therapeutic phenytoin levels as levels are often drawn once a day, leaving the possibility that breakthrough seizures may occur during an uncaptured time when phenytoin levels are subtherapeutic. Data regarding intravenous (IV) versus enteral phenytoin use were not collected; however, patients were typically started on IV phenytoin and transitioned to enteral phenytoin to complete the 7-day prophylactic course at the discretion of the primary team. Patients with early PTS had a shorter phenytoin duration due to switching to a different AED once a seizure occurred. Patients were categorized as having seizures only if this was confirmed on a cEEG, which may underestimate the number of seizures. The use of gabapentin, which may have anti-epileptic properties though relatively weak, was more frequent in the no PTS group compared to the PTS group. Patients with more severe TBI and PTS generally do not have an indication for gabapentin, while gabapentin is often used to manage neuropathic pain and/or alcohol withdrawal in patients with less severe TBI. Other AEDs have been studied for early PTS prophylaxis; however, these agents were not included in this study. Despite a higher proportion of early PTS observed in this study, the absolute number of PTS was low; this limited the ability to conduct robust multivariate analysis to identify independent risk factors for early PTS.

This study showed that patients with a higher Marshall score, age, and neurosurgical procedures for hematoma evacuation had higher incidences of breakthrough early PTS despite the use of phenytoin prophylaxis. The majority of patients had therapeutic phenytoin levels at the time of PTS. Further studies are warranted to confirm factors associated with breakthrough early PTS and to determine the optimal monitoring and therapeutic phenytoin targets to prevent early PTS.

## Data availability statement

The raw data supporting the conclusions of this article will be made available by the authors, without undue reservation.

## Ethics statement

The studies involving humans were approved by Stanford Institutional Review Board. The studies were conducted in accordance with the local legislation and institutional requirements. Written informed consent for participation was not required from the participants or the participants’ legal guardians/next of kin in accordance with the national legislation and institutional requirements.

## Author contributions

EG: Conceptualization, Data curation, Investigation, Methodology, Writing – original draft, Writing – review & editing. CD: Conceptualization, Data curation, Investigation, Methodology, Project administration, Writing – original draft, Writing – review & editing. CH: Conceptualization, Data curation, Investigation, Methodology, Writing – original draft, Writing – review & editing. ER: Conceptualization, Data curation, Investigation, Methodology, Writing – original draft. RR: Formal analysis, Methodology, Writing – original draft, Writing – review & editing. NL: Data curation, Writing – original draft. LR-L: Conceptualization, Data curation, Investigation, Methodology, Writing – original draft, Writing – review & editing.
