# Supplementary material for: Assessing risk factors associated with breakthrough early post-traumatic seizures in patients receiving phenytoin prophylaxis
Source: Front Neurol. 2024 Jan 4;14:1329042. doi: 10.3389/fneur.2023.1329042 (PMC10795534; doi:10.3389/fneur.2023.1329042)
Supplement: Supplementary file 1 [file Data_Sheet_1.pdf]

Supplemental Materials

Table of Contents **Page**

1. Secondary outcomes definitions..……………………………………………….……...1
2. ICD9/10 codes ………………………………………………………………………1
3. Supplemental methods and definitions………………………………………………1-2
4. Supplemental Table 1: Seizure data…………………………………………………3
5. Supplemental Table 2: Univariate logistic regression analysis of potential risk factors associated with early post-traumatic seizures.…...3
6. Supplemental Table 3: Bivariate binary logistic regression analysis for early post traumatic seizure including age and Marshall score ……..3
7. Supplemental Table 4: Bivariate binary logistic regression analysis for early post traumatic seizure including age and hematoma evacuation ……………………………...3

Secondary outcomes definitions

Outcome	Definition
Epileptiform activity on the continuous electroencephalograph (cEEG)	Based on EEG report from epileptologist interpretation within electronic medical record (EMR)
Immediate phenytoin level prior to seizure	Available level prior to reported time of seizure (adjusted for hypoalbuminemia and renal function)
In-hospital seizure after 1 week	As documented clinical seizure within the EMR
ICU length of stay	Number of days spent in the ICU
Hospital length of stay	Number of days spent in the hospital
In-hospital mortality	As documented within the EMR

ICD-9/10 codes

S02, S03, S06, S07, 800, 801, 802, 803, 804, 852.2, 854, 951.9

Supplemental methods and definitions:

Inclusion criteria

1. Rationale to only include patients started on phenytoin for seizure prophylaxis within 24 hours of admission for at least 6 days or until the first hospital seizure:
   a. At least 6 days – to be able to assess occurrence of early seizures while on phenytoin prophylaxis. We chose 6 days cut off rather than 7 as phenytoin orders may be discontinued slightly early.
   b. First hospital seizure – Phenytoin prophylaxis is often discontinued and an alternative AED is initiated when a seizure occurs. This may be thought to be failure of phenytoin seizure prophylaxis.

Phenytoin levels

1. **Appropriately drawn phenytoin maintenance level** was defined as a level drawn 6-8 hours following a dose to reflect a trough level. Maintenance phenytoin level typically dosed every 8 hours. A window of 6-8 hours was chosen as levels may be drawn early or late in practice.
   Winter ME. *Basic Clinical Pharmacokinetics*. 5th edition. Philadelphia, PA: Lippincott Williams & Wilkins; 2010.
2. **Inappropriately drawn phenytoin maintenance level** was defined as a level drawn within 6 hours of a dose or greater than 8 hours following a dose.
3. **Percent time in therapeutic range** was calculated for each patient with at least 1 appropriately drawn maintenance level as: (number of appropriately drawn therapeutic levels [10-20 mcg/dL]) / (total number of appropriately drawn levels)

Marshall score

Category	Definition
Diffuse injury I	No visible intracranial pathology seen on CT
Diffuse injury II	Cisterns are present with midline shift 0-5mm and/or lesion densities present no high- or mixed-density lesion >25mL; may include bone fragments and foreign bodies
Diffuse injury III	Cisterns compressed or absent with midline shift 0-5mm, no high- or mixed-density lesion > 25mL
Diffuse injury IV	Midline shift > 5 mm, no high- or mixed-density lesion > 25mL
Diffuse injury V (evacuated mass lesion)	Any lesion surgically evacuated
Diffuse injury VI (non-evacuated mass lesion)	High- or mixed-density lesion > 25mL, not surgically evacuated

Marshall LF, Marshall SB, Klauber MR, Van Berkum Clark M, Eisenberg H, Jane JA et al. The diagnosis of head injury requires a classification based on computed axial tomography. *J Neurotrauma*. 1992;9 https://globalneuro.org/uploads/files/Marshall_CT_Score_paper_abstract.pdf.

Stratification of Marshall score > 2 was based on prior literature showing that a score > 2 was associated with increased mortality.
Munakomi S. A comparative study between Marshall and Rotterdam CT scores in predicting early deaths in patients with traumatic brain injury in a major tertiary care hospital in Nepal. Chin J Traumatol. 2016;19(1):25-7. doi: 10.1016/j.cjtee.2015.12.005. PMID: 27033268; PMCID: PMC4897827.

Supplemental Table 1: Seizure data

	Frequency (n = 14)
**Early post-TBI seizure, n (%)**	
**Focal**	13 (93%)
**Generalized**	1 (7%)
**Epileptiform activity on cEEG, n (%)**	
**Status epilepticus**	5 (36%)
**Clinical**	1 (7%)
**Subclinical seizures**	8 (57%)

Seizure classification was based on neurologist notes documented in the electronic medical record.

Supplemental Table 2: Univariate logistic regression analysis of potential risk factors associated with early post-traumatic seizures

Variable	Odds Ratio (95% CI)	p-Value
**Age**	1.04 (1.01 – 1.07)	0.01
**Marshall score**	1.7 (1.15 – 2.61)	0.02
**Marshall score > 2**	6.4 (1.68 – 24.77)	0.01
**Number of Neurosurgery procedures**	1.95 (1.09 – 3.49)	0.05
**Hematoma evacuation**	5.80 (1.78 – 18.94)	0.02

Supplemental Table 3: Bivariate binary logistic regression analysis for early post traumatic seizures including age and Marshall score > 2

Variable	Odds Ratio (95% CI)	p-Value
**Age**	1.05 (1.01 – 1.08)	0.01
**Marshall score > 2**	7.44 (1.80 – 30.83)	0.01

Supplemental Table 4: Bivariate binary logistic regression analysis for early post traumatic seizures including age and hematoma evacuation

Variable	Odds Ratio (95% CI)	p-Value
**Age**	1.04 (1.01 – 1.07)	0.02
**Hematoma evacuation**	5.0 (1.46 – 17.11)	0.01
